# Association between Adult Stature and Energy Expenditure in Low-Income Women from Northeastern Brazil

**DOI:** 10.1371/journal.pone.0131891

**Published:** 2015-07-06

**Authors:** Fabiana Cristina Alves Albuquerque, Nassib Bezerra Bueno, Ana Paula Grotti Clemente, Eduardo Ferriolli, Telma Maria Menezes Toledo Florêncio, Daniel Hoffman, Ana Lydia Sawaya

**Affiliations:** 1 Department of Nutrition, Federal University of Alagoas, Maceió, Alagoas, Brazil; 2 Department of Clinical Medicine, University of São Paulo, Faculty of Medicine of Ribeirão Preto, Ribeirão Preto, São Paulo, Brazil; 3 Department of Physiology, Federal University of São Paulo, São Paulo, Brazil; 4 Department of Nutritional Sciences, Rutgers University, Newark, New Jersey, United States of America; University of Alabama at Birmingham, UNITED STATES

## Abstract

**Background:**

Perinatal undernutrition may lead to important metabolic adaptations in adult life, short stature being the most visible. The present study aimed to evaluate the association between stature and total energy expenditure of low-income women.

**Method:**

Women aged 19–45 years from low-income communities in Maceió-AL were recruited. A sample of 67 volunteers was selected and divided into either short stature (≤152.4 cm; n = 34) or non-short stature (≥158.7 cm; n = 33) group. Data on socioeconomic status, anthropometric variables, and hormonal profiles was collected. Total energy expenditure and body composition were assessed by the doubly labeled water technique with multiple points over 14 days. In addition, physical activity levels were measured with triaxial accelerometers and dietary intake data were collected using three 24-hour food records.

**Results:**

The mean subject age was 30.94 years. Women of short stature had lower body weight and lean body mass compared to non-short women, but there were no differences in thyroid hormone concentrations or daily energy intake between the two groups. Short-stature women showed lower total energy expenditure (P = 0.01) and a significantly higher physical activity level (P = 0.01) compared to non-short women. However, the difference in total energy expenditure was no longer significant after statistical adjustment for age, lean body mass, and triiodothyronine concentrations.

**Conclusion:**

Women with short stature present the same energy intake, but lower total energy expenditure than non-short women, even with a higher physical activity level, which suggests that they are more prone to weight gain.

## Introduction

Dietary and anthropometric changes related to the nutrition transition in developing countries have been occurring at an accelerated pace as the prevalence of undernutrition has decreased and that of obesity has increased. However, there remains a relatively high prevelance of chronic undernutrition, manifested as short stature, in many transitional countries, and it has been reported that short stature is associated with obesity, especially in low-income populations [1–3]. In Brazil, given the extent of the social contrasts, some populations are at a much greater risk for undernutrition and short stature, a cumulative effect of poverty that lingers for various families’ generations [2,4–6]. However, the precise reason that short stature is associated with obesity remains unclear, the primary focus of this study.

Previous studies have reported that an increased prevalence of overweight rates in many low-income populations, such as those in northeastern Brazil. For example, Florencio et al. [7] found the coexistence of short stature (22%) and overweight (25%) in poor female residents of shantytowns and low-income neighborhoods. In addition, Barbosa et al. [8] reported that within a cohort of short-stature adults, 41.2% had excess weight while 5.9% were classified as undernourished. The coexistance of under- and overnutrition is also found in other states of Brazil. In São Paulo, Sawaya et al. [9] reported a study of 535 families residing in slums and found that 8.5% of the adults suffered from undernutrition and 36.5% were overweight.

A potential explanation for the observations that short stature is associated with obesity begins with the sequela of physiological changes following undernutrition in perinatal life. We reported previously that early undernutrition leads to reduced energy requirements and central nervous system modifications that may facilitate fat accumulation [10,11]. Also, it has been found that stunted adolescent girls show lower basal metabolic rates (BMR) than their non-stunted counterparts [12]. Hence, these individuals would be more prone to remaining in a positive energy balance when faced with an increased availability of food, which could explain the findings that food consumed does not account for the high prevalence of obesity in some studies [13].

Total energy expenditure (TEE) is composed of BMR, the thermic effect of food, and physical activity level (PAL). The latter is the most variable component of energy expenditure and may be assessed using motion sensors similar to accelerometers. In turn, the gold standard method for assessing TEE is the doubly labeled water (DLW) method [14–15], a technique that allows for free-living assessment of TEE as it does not interfere with daily activities [16]. However, the use of this technique in epidemiological studies for estimating TEE is relatively rare owing to the high methodological costs involved [17]. Some studies have been conducted in Brazil, but neither in low-income populations nor to investigate the relationship bewteen height and TEE [18–20].

Obesity is a serious public health concern and the increasing prevalence of obesity in Brazil, especially in low-income communities, remains a scientific and public health priority. Given that low-income individuals with short stature are likely to present with metabolic adaptations, such as reduced energy expenditure, improving the understanding of how short stature may contribute to the risk of obesity is important. Therefore, the primary of aim of this study was to evaluate the TEE of low-income women living in peripheral areas of the city of Maceio in northeastern Brazil and determine the relationship between stature and parameters of energy expenditure.

## Materials and Methods

### Ethical aspects

All participants provided their written consent. The study and the consent procedure were approved by the Ethics Committee in Research of the Centro de Estudos Superiores de Maceió under protocol number 1588/12 in accordance to the principles expressed in the Declaration of Helsinki.

### Local

This study was conducted at the Center for Nutritional Recovery and Education—(CREN-AL) Maceió-AL. CREN is a non-governmental organization with the objective of capturing the nutritional status of malnourished children as an extension program of the Federal University of Alagoas. Analysis of stable isotopes to estimate energy expenditure and body composition data was performed at the Faculty of Medicine of Ribeirão Preto, University of São Paulo.

### Sample and study design

This cross-sectional study consisted of 100 low-income women 19–45 years old with ties to the CREN. As our objective was to compare a group of adult women with short stature, which may be a consequence of chronic undernutrition, with a group without short stature, the sample was categorized according to height. The short stature group was composed by women shorter than 152.4 cm (5^th^ percentile of the growth curves from the World Health Organization [WHO]) [21], and the non-short stature group was composed by women taller than 158.7 cm (25^th^ percentile of the same growth curves) [21]. Although the 25^th^ percentile is still below of what is expected for a group with average stature, it is noteworthy that in the population studied, few individuals are at or above the 50^th^ percentile. Pregnant women and those with physical disabilities that interfere with anthropometry or energy expenditure were excluded from the analysis.

### Socioeconomic and anthropometric evaluation

With the objective of characterizing the sample, a standardized form was utilized to collect socioeconomic, anthropometric, and lifestyle data at CREN. The measurement of anthropometric data followed the recommendations of Lohman et al. [22]. To obtain weights, a digital scale (Filizola, São Paulo) with a minimum capacity of 1 kg and maximum capacity of 150 kg was used. Height was measured using a wall stadiometer with a minimum capacity of 300 mm and a maximum capacity of 2,000 mm in 1-mm increments. The current nutritional status of these adults was obtained using the body mass index (BMI), normal being values ≥18.5 but <25 kg/m^2^ and overweight/obese being values ≥25 kg/m^2^ [23].

Measurements of waist and hip circumference were collected using a tape measure with the women standing. Waist circumference was obtained at the midpoint of the distance between the last rib and the anterior superior iliac spine, while hip circumference was obtained in the region of greater circumference between the waist and thigh according to WHO recommendations [23].

### Dietary intake data

Dietary intake was assessed by using the 24-hour food record method on three different days (one on the weekend). A trained nutritionist applied the questionnaires with the aid of a photographic book and performed the coding and analysis of dietary assessments [24].

### PAL

To evaluate the PAL, activPAL (Glasgow, UK) triaxial accelerometer type motion sensors were used. These tools measure acceleration in the anteroposterior, lateral, and vertical body axes to monitor physical activity performed by the women in their daily activities. The accelerometers were placed on the women’s thighs for 7 days. The women were asked to not remove them until the deadline, but were allowed to shower and perform any activities except submerging them in water.

### Biochemical tests

The participants were instructed to fast for 12 hours and had blood samples obtained by venipuncture performed by a trained team member. Thyroid-stimulating hormone, triiodothyronine, and thyroxine levels were measured.

### TEE and body composition

For the measurement of TEE, the multiple-point DLW technique for a period of 14 days was used [25,26]. The data obtained were used as a reference for the energy requirements for the production rate of CO_2_, which is equal to the difference between the elimination rates of isotopes and corrected for whole body water [16,27]. To participate in the the DLW assessment, the volunteers could not be febrile and should not have practiced intense workouts the day before or received intravenous fluids within the previous week. Furthermore, they could not move out of Alagoas during the 14 days after administration to avoid a shift in the water consumed.

DLW was calculated according to Schoeller [28] and Schoeller et al. [29] as the average weight of the sample (67 kg). On day 1, a non-enriched urine sample was collected from each volunteer and then a dose of DLW was completely consumed, and the time of consumption was noted. After 30 minutes, the subjects consumed a standard breakfast of fruit juice, crackers, fruit, and cake. Subsequent urine samples were collected once a day at the same time in the volunteers’ homes on the 1^st^, 2^nd^, 3^rd^, 7^th^, 12^th^, 13^th^, and 14^th^ days after the administration of the dose. All samples were refrigerated until analyzed.

The samples were transported to the Department of Clinical Medicine, University of São Paulo—Ribeirão Preto and prepared for isotopic weighing (^2^H/^1^H and ^18^O_2_/^16^O_2_) and analysis using the mass spectrometry isotope ratio (Hydra System HIP 20–20; Europa Scientific, Cheshire, UK). The analysis was performed according to a previously established protocol [30]. Quality controls were employed to ensure proper analysis, and all dosages fulfilled the following criteria: the ratio between the ^2^H dilution space and the ^18^O_2_ dilution space should be 1.01–1.06 [30] and differences in triplicate samples should be 5 deltas to ^2^H and 0.5 delta to ^18^O_2_ [31].

For the hydrogen isotope analysis, a platinum catalyst that promotes equilibrium between the hydrogen from water with the hydrogen injected into the tube was employed. Three 0.5-mL urine samples were placed in vacutainers with small sticks of platinum catalysts. The same volume used for the standards was used for a sample of unlabeled drinking water and diluted dose of the DLW, with a proportional dilution similar to the dose administered to the subjects and the urine samples. The vacutainers were allowed to stand for 6 hours [32]. For the measurement of ^18^O, three 0.5-mL urine samples were filled with 3% CO_2_ and allowed to stand for 24 hours to achieve liquid–gas balance [33–34].

For body composition assessment, lean body mass (LBM) and fat mass (FM) were derived from the total body water, calculated using the dilution space of the ^2^H analyses [35]. Resting energy expenditure (REE) was estimated using LBM in the following equation: REE (kcal) = 1641 + [91.3 x LBM (kg)], based on the work by Nelson et al. [36]. An estimate of energy expenditure for physical activity was calculated as the difference between TEE and REE, assuming that 10% of the TEE is due to postprandial thermogenesis.

### Statistical analyses

Continuous variables are presented as means and standard deviations, while categorical variables are presented as absolute and relative frequencies. These values were compared through Fisher's exact test. Continuous variables were subjected to Levene’s test to verify the assumption of variance homogeneity. When this assumption was met, comparisons were undertaken by the t-test for independent samples. When this was not the case, the Welch’s t-test was used.

To further explore the relationship between TEE and short stature in adults, an analysis of covariance (ANCOVA) was conducted to determine the association between TEE and height while controlling for potential confounding factors, including age (years), LBM (Kg), and triiodothyronine concentrations. These specific factors were included in the analysis as they are independent predictors of total energy expenditure [37,38]. We did not include any physical activity measure, such as PAL, in the model as physical activity is not a predictor of TEE, but is an integral component of TEE. Also, while dietary intake is associated with TEE, it is not a a true confounding variable as it may be part of the causal relationiship between short stature and energy expenditure. Moreover, the methods used to assess dietary intake are generally much less accurate relative to using DLW to estimate TEE. The assumption of homogeneity of regression slopes between the covariates was tested. We adopted an α value of 5% for all analyses. The analyses were conducted using the Statistical Package for Social Sciences v20.0 (IBM Inc., Chicago, IL, USA).

## Results

The socioeconomic characteristics of the study sample are shown in Table 1. The sample consisted of young women (mean age, 31 years). In the household of each of the women there was an average of 5 people and the families presented a per capita monthly income of 68.8 USD for the short stature group and 87.35 USD for the non-short group, approximately 2.30 and 2.90 USD per day, respectively, without a significant difference. A sewage system was present for 90.3% of the homes of non-short women and 66.7% of the homes of women with short stature (p = 0.02). Mean schooling years were 5.63 and 7.87, respectively, ranging from functional illiteracy to complete high school, among women with short stature and non-short women, respectively (p = 0.009).

**Table 1:** Socioeconomic data of the sample.

Variable	Short stature (n = 34)		Non-short (n = 33)		P-value
	Mean or “n”	Standard deviation or %	Mean or “n”	Standard deviation or %	
Age (years)^1^	31.35	4.93	30.55	5.77	0.391
Schooling (years)^1^	5.63	3.14	7.87	3.45	0.009
Number of family members^1^	5.12	1.53	4.58	1.45	0.154
Per capita income (R$)^1^	187.79	107.35	235.86	167.48	0.174
Receive government benefits^2^	29	87.9	26	83.9	0.729
Housewives^2^	22	66.7	18	58.1	0.607
House has: ^2^					
Water supply by well^2^	32	97	28	90.3	0.347
Sewage system^2^	22	66.7	28	90.3	0.033
Coated walls^2^	22	66.7	28	60.0	0.611
Coated floors^2^	12	36.4	15	48.4	0.448

^1^Results expressed as mean and standard deviation.

^2^Results expressed as absolute and relative frequency.

Anthropometric data are summarized in Table 2. The majority of the sample was overweight with high adiposity according to BMI variables and body fat percentages, respectively. The women of short stature had significantly lower body weight (p = 0.011), height (p < 0.001), and LBM (p < 0.001) than the non-short women, however, there was no difference in BMI (p = 0.248). It is noteworthy that, despite being shorter, women of short stature did not have a lower fat mass (kg) than non-short women. There were no differences in thyroid hormone concentrations or dietary intake data. The metabolic parameters obtained by the DLW technique used to calculate the TEE are provided in Table 3.

**Table 2:** Anthropometric, biochemical, and dietary data of short and non-short subjects.

Variable	Short stature (n = 34)		Non-short (n = 33)		P value^1^
	Mean	Standard Deviation	Mean	Standard Deviation	
Weight (kg)	63.23	11.84	71.47	13.84	0.011
Height (cm)	148.95	3.63	162.72	3.30	<0.001
BMI (kg/m^2^)	28.43	4.67	27.01	5.39	0.248
Waist circumference (cm)	86.45	12.80	86.77	14.08	0.923
Hip circumference (cm)	101.47	8.87	104.12	11.22	0.285
Lean body mass (kg)	35.20	4.69	41.30	6.04	<0.001
Fat mass (kg)	28.02	9.68	30.17	13.75	0.461
Body fat (%)	43.42	6.87	40.44	13.78	0.270
Energy intake (kcal/day)	1,799.1	439.6	1,899.6	472.6	0.370
Carbohydrate intake (g/day)	255.7	77.2	263.2	80.8	0.697
Lipid intake (g/day)	52.7	13.6	58.6	16.8	0.121
Protein intake (g/day)	72.6	21.3	74.6	22.1	0.701
Free Triodothyronine (ng/dL)	1.24	0.204	1.26	0.287	0.655
Free Thyroxin (ng/dL)	9.37	1.69	9.44	2.44	0.888
Thyroid-stimulating hormone (mUI/L)	1.69	0.839	1.80	1.43	0.691

^1^P value on t test of independent samples.

**Table 3:** Metabolic parameters from the doubly labeled water protocol.

Variable	Mean	Standard deviation	Median	Minimum	Maximum
Total energy expenditure (kcal/day)	2,186	504	2,156	1,064	4,073
CO_2_ production rate (mol/day)	17.2	4.0	17.0	7.9	32.0
Nd (mol)	1,588.0	314.0	1,564.7	790.9	2,795.6
No (mol)	1,662.9	325.2	1,634.7	843.0	2,870.6
Nd/No	1.05	0.02	1.04	1.02	1.09
Kd (mol/dia)	0.1207	0.0158	0.1193	0.0941	0.1516
Ko (mol/dia)	0.0922	0.0146	0.0907	0.0662	0.1231

Nd, deuterium dilution space; No, oxygen-18 dilution space; Nd/No, ratio between dilution spaces; Kd, deuterium disposal rate; Ko, oxygen-18 disposal rate

Results from analyses of data on parameters of enegy expenditure are summarized in Table 4. The short women had a lower TEE compared to the non-short women (2,041 v. 2,331, respectively, p = 0.016). Phhysical activity assessed using an accelerometer was higher in the short women compared to the non-short women (1.49 v. 1.45 ± 0.05, respectively, p = 0.012). However, there were no differences between the groups when energy expenditure from physical activity was estimated from the body composition and TEE estimates (p = 0.21).

**Table 4:** Parameters of energy expenditure for short and non-short subjects.

Variable	Short stature (n = 34)		Non-short (n = 33)		P value
	Mean	Standard Deviation	Mean	Standard Deviation	
Total energy expenditure (kcal)	2,041	430	2,331	536	0.016
Resting energy expenditure (kcal)	1,159	102	1,292	131	<0.001
Energy expenditure from physical activity (kcal)	677	350	809	493	0.21
Physical activity level (METs/24hours)	1.49	0.06	1.45	0.05	0.012
Adjusted analysis	Mean	95% CI	Mean	95% CI	P value
Total energy expenditure (kcal)*	2,098	1,919–2,278	2,276	2,094–2,459	0.19

*adjusted for age, body composition, and hormonal profile.

When TEE was corrected for age, LBM, and triiodothyronine concentrations (Table 4B), differences in TEE between the height categories was not statistically significant (p = 0.19), being 2,098 kcal/day for women in the short group compared to 2,276 kcal/day for women in the non-short group.

## Discussion

As the prevalence of obesity continues to increase in many transitional countries, is it of public health and scientific importance to understand how poor growth, another public health problem in these countries, contributes to obesity [1–3]. Briefly, we found that women of short stature had lower TEE and LBM compared to non-short women from the same low-income communities. The differences in TEE were not statistically significant when adjusted for body composition and hormonal profile. However, the fact that estimated energy intake was not different between the two groups suggests that the shorter women may be at greater risk for positive energy balance, a major contributor to obesity.

To our knowledge, this is the first study to investigate the relationship between stature and parameters of energy expenditure in adults. Our findings are consistent with previous investigations of short stature and TEE in children [39] and short stature and REE in adolescents [40]. As with our data, these studies found that controlling for the primary predictor of TEE, metabolically active tissue (i.e. LBM), attenuated the relationship between TEE and height. There is some concern that statistically controlling for LBM to determine the independent relationship between height and energy expenditure may introduce bias into the analyses, given that LBM is not only associated with TEE, but also with height. However, the collinearity between LBM and TEE is greater than what is reported for LBM and height and the physiological relationship is direct [41]. Thus, the use of LBM as a co-variate in the statistical analysis of stature and energy metabolism is not only acceptable, but also warranted. Given the consistency of findings across the lifespan, it is evident that physiological changes that may contribute to positive energy balance persist into adulthood.

In terms of specific aspects of energy expenditure that may be influenced by poor growth, Hoffman et al. [19] reported that stunted children had a lower BMR and, significantly lower rate of fat oxidation (i.e. higher RQ) compared to normal height children, factors that could predispose short-stature individuals to obesity compared to their non-short counterparts [13]. Furthermore, a low BMR can lead to a decreased TEE, which could justify our findings of lower TEE in women of short stature. Disse-Mohamed et al. [42] also assert that individuals who have undergone dietary deprivations during the first years of life tend to present a reduced lipid oxidation rate, a risk factor for body fat accumulation. Perhaps more important, a low rate of fat oxidation will promote fat gain when accompanied by an increased fat intake and/or low level of physial activity.

Regarding physical activity, the short-stature women presented higher PAL compared to the non-short women when assessed with an accelereomter, but the energy expenditure for physical activity was not different between groups when assessed using DLW and a prediction equation for BMR. The disparate results presented are most likely due to methodological differences in exactly what is measured by each methodology, such as differences in estimated CO_2_ production over a 14-day period with DLW or discrete movements over a short period with the accelerometer. While both methods are accurate in terms of estimating activity, it is difficult to compare the two given the inherent methodological differences. Regardless, a higher PAL may provide a protective factor against weight gain, but more intricate research is required to more fully address this hypothesis.

In terms of how our results compared to other studies, it is important to note that few studies have used the DLW method to understand how poor growth influences energy expenditure. In those studies that have addressed this point, all studied undernourished children, adults of high socioeconomic status, or the elderly [18–20]. Scagliusi et al. [18] found an average TEE of 2,622 kcal/day in women with a mean age of 34 years. Although their sample was similar to ours, per capita income and TEE were substantially lower in the present study, consistent with other studies in Brazil suggesting that the higher the income, the greater the PAL [43,44]. In a study by Ferriolli et al. [20], women with a mean age of 66 years presented an average TEE of 2,154 kcal/day, similar to the average value found in the present study (2,186 kcal/day) of women with a mean age of 31 years. After stratifying our data by height, this result is even more significant since women of short stature have a TEE of only 2,042 kcal/day.

There are a few limitations of the present study that merit discussion. First, the absence of an objective measure of BMR, such as indirect calorimetry, to determine specific reasons to account for differences in TEE between groups was not available. However, we were able to estimate BMR using an accurate technique of body composition, such as ^2^H dilution. Second, our control group consisted of women with stature that was below the average of growth references, a factor that could influence the comparability with other populations, but do not invalidate the differences observed. The specific reason that cutoff used was chosen was primarily due to the fact that there are very few women in the community studied who are taller than the 50^th^ percentile. Still, we were able to find significant differences in TEE between groups. Third, dietary intake was assessed via self-report which has an accuracy of 60–70% of daily energy requirements [18]. Finally, it is also important to note that the cross-sectional nature of the study does not allow solid conclusions to be made that the lower TEE will result in weight gain for the short-stature group.

In summary, given the significant public health impact of the “double burden” in transitional countries, it is important to improve the understanding behind the relationship between poor growth early in life and later risk for obesity. The results of our study suggest a potential explanation for why adults who are short are more likely to be overweight or obese, given the difference in TEE, but not energy intake, in the cohort studied in a low-income community. Nonetheless, longitudinal studies are warranted to fully understand the mechanisms of these observations and to determine wheter or not shorter women, in fact, gain more body fat compared to women who are taller.

## Supporting Information

S1 File Database. (XLSX)
